# Mid-term outcomes of the R3™ delta ceramic acetabular system in total hip arthroplasty

**DOI:** 10.1186/s13018-020-02192-6

**Published:** 2021-01-09

**Authors:** Edward T. Davis, Ville Remes, Petri Virolainen, Peter Gebuhr, Bart Van Backlé, Matthew P. Revell, Branko Kopjar

**Affiliations:** 1 The Royal Orthopaedic Hospital, NHS Foundation Trust, Bristol Road South, Northfield, Birmingham, B31 2AP UK; 2 Department of Orthopedics, HUS Peijaksen sairaala, P.O. Box 900, 00029 HUS, Vantaa, Finland; 3 Turku University Hospital, Kiinamyllynkatu 4-8, P.O Box 52, 20521 Turku, Finland; 4 Orthopaedic Surgeon, Department of Orthopaedics, Hvidovre University Hospital, Copenhagen, Denmark; 5 AZ Nikolaas, Sint Niklaas, Regentiestraat, 60, 9100 Sint Niklaas, Belgium; 6 Department of Health Services, University of Washington, H690C, Health Sciences Building, P.O. Box 357660, Seattle, WA 98195-7660 USA

**Keywords:** Bearing, Ceramic-on-ceramic (CoC), Complications, Delta ceramic, Modified Harris hip score (mHHS), Outcomes, R3™ delta Ceramic Acetabular System, Total hip arthroplasty (THA), UCLA Activity Rating Scale (UCLA ARS), Western Ontario and McMaster Universities Osteoarthritis Index (WOMAC®)

## Abstract

**Background:**

Whilst bony fixation of hip replacement has stable solutions, there remains controversy over which bearing best optimizes longevity and function. Ceramic-on-ceramic (CoC) bearing combinations are associated with lower risk of revision due to aseptic loosening and dislocation. Evidence for long-term functional outcomes of modern, 4th generation CoC bearings is limited. The aim of this study was to analyze outcomes and complications of the R3™ Acetabular System (Smith & Nephew, Inc., Cordova, TN, USA) in combination with BIOLOX® Delta ceramic femoral head in patients undergoing primary total hip arthroplasty (THA).

**Methods:**

Between June 2009 and May 2011, 175 patients (178 hips) were enrolled into a prospective, study at 6 sites in Europe and prospectively followed-up at 3 months and 1, 3, 5, and 7 years postoperative.

**Results:**

Total WOMAC score improved from 63 (range, 22–91) preoperative to 8 (range, 0–8) at 1-year follow-up and remained unchanged at 7-year follow-up. Modified Harris hip score improved from 45 (range, 10–87) preoperative to 83 (range, 25–100) at 3 months, 91 (range, 42–100) at 1 year, and 92 (range, 46, 100) at 7 years. UCLA Activity Rating Scale score improved from 3.3 (range, 1–8) preoperative to 6.2 (range, 2–8) at 1 year; it marginally declined to 5.8 (range, 3–8) at 7-year follow-up. There were 4 trochanteric fractures and 5 patients died of unrelated reasons. Three hips were revised (2 periprosthetic fractures and 1 subluxation). The 7-year cumulative survival rate was 98.3%.

**Conclusion:**

Clinical and functional improvements of THA with CoC bearing are maintained at 7 years postoperative.

**Trial registration:**

ClinicalTrials.Gov, NCT03566082, Registered 10 January 2018—retrospectively registered,

## Background

Total hip arthroplasty (THA) is one of the most successful orthopedic interventions [1]. Although the number of successful operations performed each year is increasing, THA is not without risk. Debris from metal-on-polyethylene (MoP) components has been associated with osteolysis, a serious complication which can lead to pain, prosthesis loosening, and periprosthetic fracture [2]. Metal-on-metal (MoM) bearings have been associated with the release of corrosion debris resulting in adverse local tissue responses [3, 4]. More recently, there has been increasing concern over the generation of metal particles from the taper junction in MoP bearings.

Ceramic-on-ceramic (CoC) bearings provide an alternative bearing choice. Although corrosion debris may still occur with ceramic bearings, the magnitude of the corrosion is less than that observed in other bearing combinations [5]. Excellent results for the CoC bearing have been published; however, long-term data is still lacking, and the CoC bearing has provided a low rate of wear which is beneficial especially in young patients.

THA using CoC articulation is not a new concept and has been used for 30 years in prosthetic hip components [6, 7]. The type of ceramic used today is aluminum oxide, also known as alumina. The improved wear characteristics of alumina ceramic may result in a longer lasting implant [6, 8, 9] and may reduce the risk of ceramic fracture compared to earlier generations of ceramic materials.

Improvements in the ceramic material have continued the evolution of ceramic bearing technology. BIOLOX® delta represents the latest advancement in alumina ceramic technology due to the addition of zirconium oxide which provides the basic hardness and wear resistance, and strontium oxide and chromium oxide which provide the improved mechanical properties. Compared with pure aluminum oxide, ceramic BIOLOX delta offers higher mechanical properties including higher fracture toughness [10].

The R3™ BIOLOX delta Ceramic Acetabular System (Smith & Nephew, Inc., Cordova, TN, USA) is a CoC hip prosthesis consisting of a CoC acetabular bearing couple combined with a compatible metal shell and one of five commercially available Smith & Nephew femoral stems.

We report the mid-term clinical and functional outcomes of the R3™ Acetabular System with a BIOLOX delta CoC bearing option in patients undergoing primary THA.

## Materials and methods

### Patients

Between June 2009 and May 2011, 175 patients (178 THAs) from a larger prospective non-randomized study were enrolled at 6 investigational sites in Europe and were followed-up in clinic at 3 months and at 1, 3, 5, and 7 years.

### Inclusion and exclusion criteria

Key inclusion criteria were ages 18–75 years, primary THA required due to non-inflammatory degenerative joint disease (e.g., osteoarthritis, post-traumatic arthritis, avascular necrosis, dysplasia/developmental dysplasia of the hip) or inflammatory joint disease (e.g., rheumatoid arthritis), and free of conditions that would pose excessive operative risk. Patients were excluded from the study if they had an active infection, sepsis, or acute hip trauma.

### Outcome measures

Clinical outcomes were assessed by the modified Harris Hip Score (mHHS) [11, 12], the Western Ontario and McMaster Universities Arthritis Index (WOMAC® 3.1) [13], and the UCLA Activity Rating Scale (UCLA ARS) [14].

Safety data collected were adverse events and device survivorship (revisions).

### Follow-up

This was a pre-planned study of 7 years duration. Five-year follow-up was available for 66.0% (115/175) of THAs and 7-year follow-up was available for 62% (106/172) THA. Unfortunately, elderly subjects are difficult to maintain in follow-up.

### Statistical analysis

Changes in scores by follow-up visit were analyzed by mixed model for repeated measures (MMRM). Post hoc comparisons with pre-surgical values were performed using Tukey adjustment. Device survivorship was analyzed by Kaplan-Meier product-limit estimator. All analyses were made using SAS/STAT® software, Version 9.4 for PC, ©2017 SAS Institute Inc., Cary, NC, USA.

### Ethical approval

All investigative sites obtained approval from their corresponding ethics committees. All patients signed an informed consent document prior to participating in the study.

## Results

### Demographics and operative characteristics

The primary preoperative diagnosis was osteoarthritis (84.7%), followed by osteonecrosis (9.5%) and dysplasia (2.9%). Operative time was 64.3 min (SD 21.3 min, range 35 to 160 minutes). A minimally invasive approach was used in 44.9% of the surgeries. An anterolateral approach was used in 70 (39.33%), transgluteal in 44 (24.72%), and posterolateral in 64 (35.96%). Average age at the time of surgery was 63.4 years (SD 9.0, range 27 to 75 years), and 65.7% were females. Average body mass index (BMI) was 28.3 (SD 5.0, range 19.0 to 44.4).

### Survival rate

**Fig. 1:**
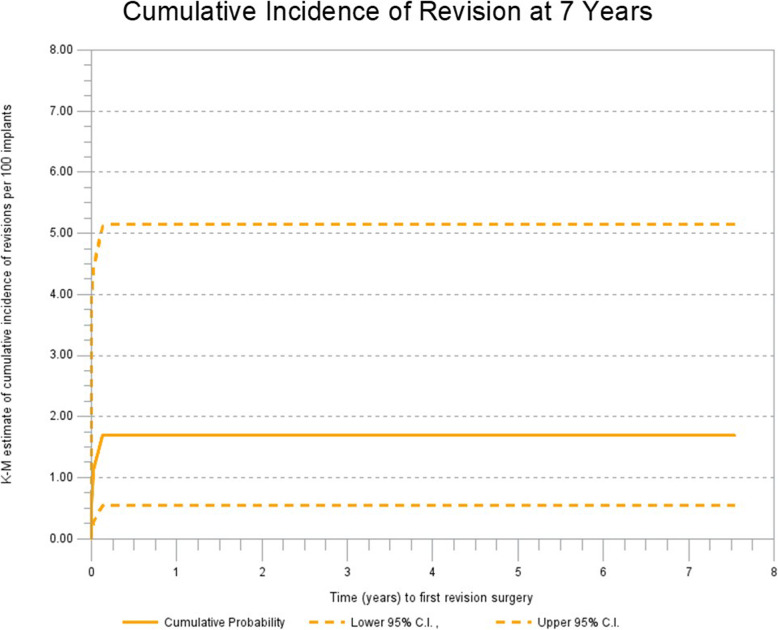
Cumulative incidence of revision at 7 years

There were three revisions, all of which occurred within 45 days of primary THA. Two revisions were due to periprosthetic femoral fracture and one due to subluxation. The cumulative incidence of revision at 7 years was 1.69% (95% C.I. 0.55 to 5.15%) (Fig. 1).

### Harris hip score

**Fig. 2:**
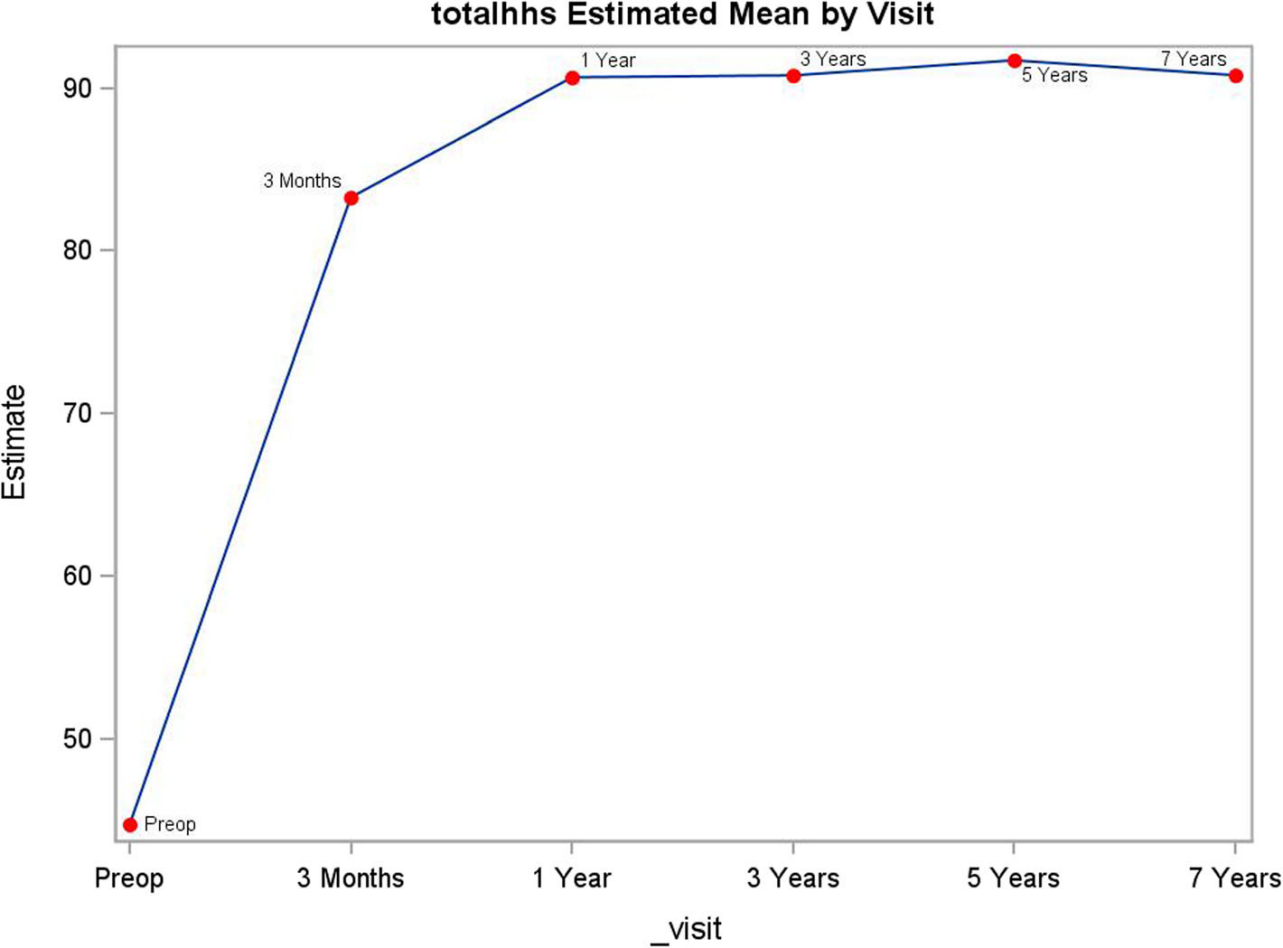
Modified Harris hip score by follow-up visit

Modified HHS improved at first follow-up visit at 3-month postoperative compared to pre-operative value (*p* < .0001). The score further improved and reached peak value at 1-year compared to 3-month follow-up (*p* < .0001). The score was maintained at last follow-up at 7 years (Table 1, Fig. 2).

**Table 1:** Clinical and patient outcomes by follow-up

		Preop	3 months	1 year	3 years	5 years	7 years
WOMAC score							
*N*		173	164	134	124	109	102
Mean		62.6	13.6	8.0	9.3	10.1	9.6
Std		15.5	11.0	11.4	11.9	13.4	11.5
Range		22, 91	0, 57	0, 57	0, 57	0, 61	0, 50
WOMAC physical function score							
*N*		174	164	134	124	110	102
Mean		45.4	10.6	6.4	7.8	8.4	8.3
Standard deviation		10.9	8.4	9.1	10.0	11.1	9.9
Range		12, 65	0, 46	0, 43	0, 46	0, 51	0, 50
WOMAC pain score							
*N*		173	164	134	124	111	102
Mean		12.2	1.7	1.1	1.1	1.3	0.8
Standard deviation		3.7	2.3	2.1	2.3	2.7	1.8
Range		3, 20	0, 11	0, 12	0, 10	0, 13	0, 9
WOMAC stiffness score							
*N*		174	164	134	124	112	102
Mean		5.0	1.3	0.6	0.4	0.7	0.5
Standard deviation		1.9	1.3	1.0	0.9	1.3	1.0
Range		0, 8	0, 6	0, 5	0, 4	0, 8	0, 5
Modified Harris hip score							
*N*		172	166	135	118	113	104
Mean		44.8	83.4	91.0	91.6	92.3	92.0
Standard deviation		12.1	13.5	12.2	12.1	11.1	11.1
Range		10, 87	25, 100	42, 100	43, 100	54, 100	46, 100
UCLA activity rating scale score							
*N*		175	167	134	124	112	102
Mean		3.3	5.7	6.2	6.1	5.9	5.8
Std		1.4	1.5	1.4	1.3	1.6	1.5
Range		1, 8	2, 8	2, 8	2,8	2, 8	3, 8

### WOMAC score

**Fig. 3:**
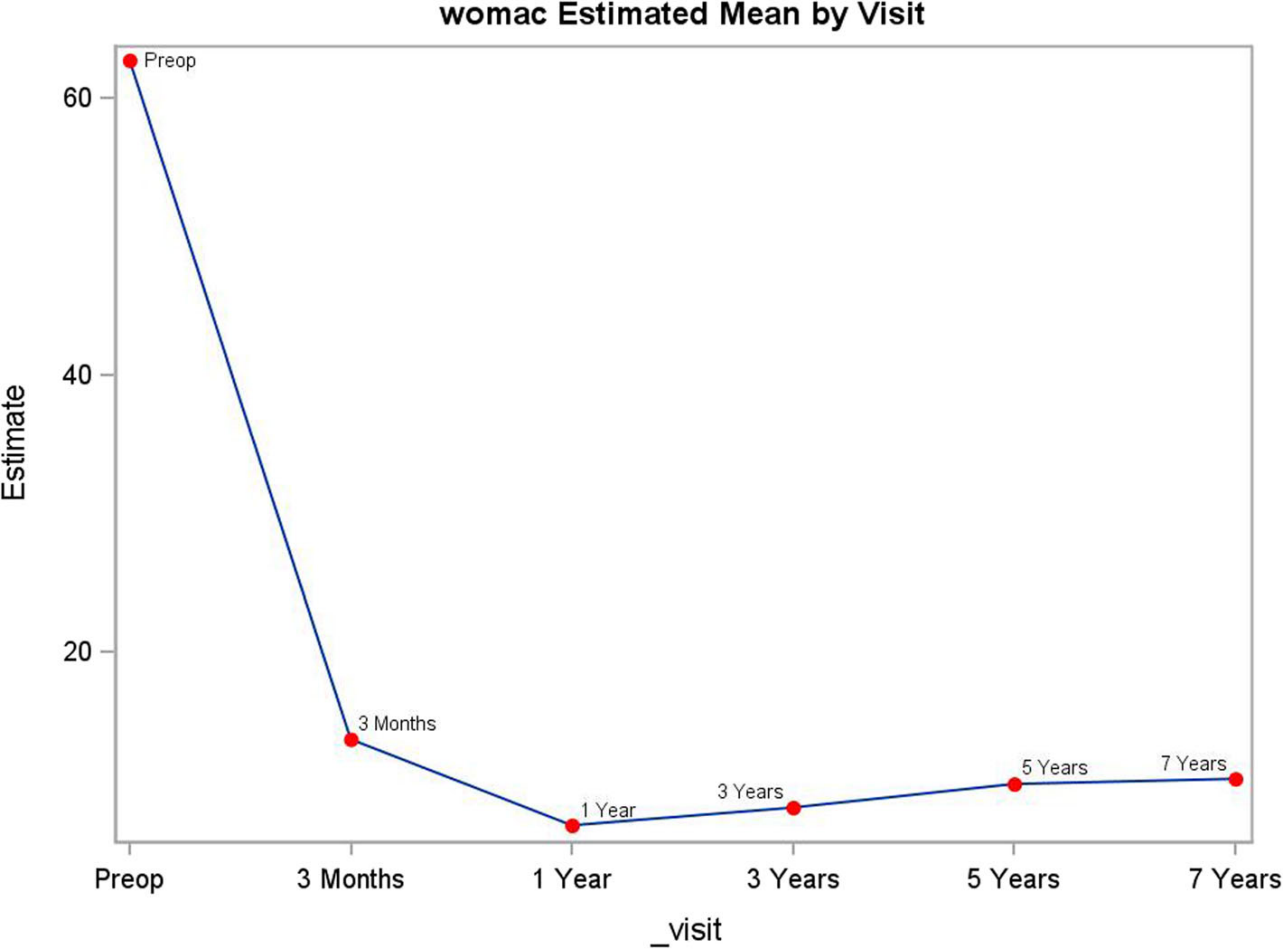
WOMAC score by follow-up visit

WOMAC score improved at first follow-up visit at 3-month postoperative compared to pre-operative value (*p* < .0001). The score further improved and reached peak value at 1-year compared to 3-month follow-up (*p* < .0001). After 1-year follow-up, there was a marginal decline in score. Statistically, the score at 7-year follow-up was lower than at 1-year follow-up (*p* = 0.0230) (Table 1, Fig. 3).

### UCLA ARS

**Fig. 4:**
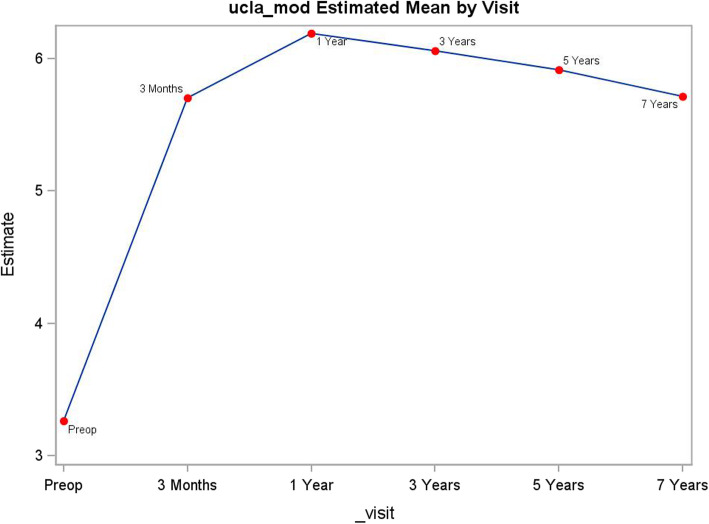
UCLA activity rating scale score by follow-up visit

The activity level measured by the UCLA ARS score improved at the first follow-up visit at 3-month postoperative compared to the preoperative value (*p* < .0001). The UCLA ARS score further improved and reached peak value at the 1-year follow-up compared to the 3-month follow-up (*p* < .0003). After 1-year follow-up, there was a marginal decline in score. Statistically, the score at the 7-year follow-up was lower than at the 1-year follow-up (*p* = 0.0032) (Table 1, Fig. 4).

### Complications

Three patients died by 5-year follow-up and an additional 2 by 7-year follow-up due to causes unrelated to the hip surgery. There were four trochanteric fractures and one superficial infection. There were no cases of breakage.

## Discussion

In this prospective study, the R3™ Acetabular System with a BIOLOX delta CoC bearing in patients undergoing primary THA resulted in favorable clinical and patient outcomes and an adequate safety profile. Compared to their pre-operative baseline, patients experienced significant and clinically relevant improvements in mHHS, UCLA ARS score, and WOMAC scores at all intervals through 7 years.

Results from previous clinical studies have evidenced the safety and functional benefits of using CoC bearing surfaces [15]. The introduction of BIOLOX delta was in an effort to make the material more resistant to fracture when compared to the BIOLOX forte compound [16]. The hard-on-hard nature of the CoC bearing has been implicated in the production of audible noise from the joint that can be described as squeaking. The instance of revision for this reason has been found to be 0.2% in the meta-analysis by Owen et al. [17] and registry data has shown revision rates of 0.1% [18].

BIOLOX delta is the 4th generation of alumina ceramic, and this generation has demonstrated increased material density and grain size five times smaller than the 3rd generation articulation [19, 20]. The unique feature of R3 ceramic liners is that they have a titanium support ring around the periphery of the liner. The support ring offers greater protection against chipped edges and tensile forces for the ceramic insert that result in high fatigue and burst performance for insert assembly [21]. Lab tests have shown that the burst strength of these liners is significantly higher than that of traditional ceramic liners with no band [22].

The revision rates in this study compare favorably with those in registry reports. The Australian Orthopaedic Association National Joint Replacement Registry [23] demonstrates a cumulative incidence of revision of CoC THA of 3.1% at 5 years and 4.9% at 10 years. The National Joint Registry of England, Wales, Northern Ireland and the Isle of Man [24] reports a cumulative revision rate for uncemented THA utilizing the CoC bearing of 2.3% at 5 years and 3.7% at 10 years. Recent analysis has shown that the increased failure rates of this combination in the NJR may be significantly affected by the rates of ceramic fracture. The high rates in the registry may be at odds with the reported revisions in this study due to the improved characteristics of the ceramic and surgeon experience in implanting this bearing combination.

In a recent study using data from the National Joint Registry of England and Wales, the CoC bearings had better survival compared to metal-on-poly (MoP) bearings but worse survival compared to ceramic-on-highly cross-linked polyethylene (CoXPE). The study however did not stratify analysis by the ceramic generation [25]. Further, the study also shows that the longevity of the prosthesis depends on the patient gender, age, head size, and stem fixation. These same factors were identified in a meta-analysis of 5321 hips [15].

Numerous recent studies have reported results that corroborate our findings. Several recent studies reported survival rates of 99.3% or higher. Buttaro et al. retrospectively reviewed 880 patients (939 hips) who underwent THA with delta CoC. There was one liner fracture, two early loosening of cups, one fracture of a femoral ball head, and one case of squeaking. Survival rate was 99.3% at mean follow-up of 5.3 years [26]. Fulin et al. evaluated 163 patients (197 hips) who underwent THA with BIOLOX delta CoC. Survival rate was 99.5% at 7.7 years for stem revision and 100% for cup revision [27]. In a prospective study of 246 patients (274 THAs) who underwent THA with delta CoC, Lee et al. reported no ceramic malseating or fracture and 8 cases of noise at 5 years. There were two revisions, one for periprosthetic fracture and one for recurrent dislocation. Survival rate was 99.6% at 8 years postoperative [28]. Kim YH et al. evaluated 277 patients (334 hips) aged 50 years or younger who underwent THA with delta CoC. Thirty-three hips had clicking and 2 had squeaking. There was no osteolysis or ceramic head or liner fracture. There was one revision. Survival rate was 99.7% at mean 7.8 years follow-up [29].

Other recent studies reported survival rates ranging from 90.5 to 98.5%. In a registry study, Castagnini et al. analyzed 327 revision hips with BIOLOX delta CoC. There were 26 re-revisions due to recurrent dislocations, aseptic cup loosening, and septic loosening. There were no ceramic fractures. Survival rate for delta CoC bearings was 90.5% at 7 years [30]. In a prospective study of 345 patients who underwent THA with delta CoC, Hamilton et al. reported 3 liner fractures and 26 cases of squeaking. There were 2 revisions for liner fracture, 4 for stem loosening, and 3 for deep infection. Survival rate was 96.9% at 6 years [31]. Baek et al. analyzed 91 patients (94 hips) who underwent THA with delta CoC. There were no ceramic fractures, but there was 1 perioperative dislocation, 2 postoperative periprosthetic fractures, and 3 cases of clicking. There were two revisions, one for liner dissociation and one for postoperative periprosthetic fracture. Survival rate was 96.8% at 5 years for reoperation and 97.9% for revision [32].

Four studies reported survival rates of 98.5% or 98.6%. Cho et al. retrospectively reviewed 242 patients (263 hips) who underwent THA with BIOLOX delta CoC. There were four revisions, one for recurrent dislocation, one for failed osteointegration, one for infection, and one for liner fracture. Survival rate was 98.5% at mean 5.2 years follow-up [33]. Luo et al. retrospectively analyzed 127 patients (135 hips) who underwent THA with BIOLOX delta CoC. There was 1 postoperative ceramic liner rim fracture and 13 hips had squeaking. Survival rate for revision was 98.5% at mean 70 months [34]. Aoude et al. analyzed 133 consecutive THAs utilizing delta CoC. There were no ceramic fractures or chipping. There were two revisions, one for infection and one for dislocation. The survival rate was 98.5% at mean follow-up of 6 years [35]. In a retrospective study of 667 patients (749 hips) who underwent THA utilizing BIOLOX delta CoC, Lim et al. reported 2 ceramic liner fractures and 48 hips with clicking or squeaking. Other complications were 1 deep infection, 1 dislocation, 3 iliopsoas tendonitis, and 6 periprosthetic femoral fractures. Survival rate was 98.6% at mean 6.5 years [36].

Lastly, Kim SC et al. performed a comparison of outcomes of 3rd-generation and 4th-generation CoC articulations in THA using registry data. Four hundred eighty-two patients (602 hips) underwent either forte (310 hips) or delta (292 hips) CoC THA. There were six dislocations in the forte group and one in the delta group. One ceramic head fracture occurred in the forte group. Clicking or squeaking occurred in 22 forte patients and 21 delta patients. There were 9 revisions, and survival rates were 98.4% and 98.6% at 5 years for the forte and delta groups, respectively [37].

The reduction in wear rates of CoC bearings when compared to polyethylene have been well documented and the relatively bioinert debris produced from the CoC bearing when compared to polyethylene debris provides the advantage of reducing aseptic loosening due osteolysis [38]. The improvements in the manufacture of ceramic have also shown a reduction in the wear rates with the BIOLOX forte compound showing steady state wear rates of 1.2 mm^3^ per million cycles, in contrast to the BIOLOX delta which demonstrated wear rates of only 0.12 mm^3^ per million cycles [39]. The mean wear rates from BIOLOX forte retrievals after a minimum of 6 months in situ were reported to be 0.6 mm^3^ per year for femoral heads and 0.5 mm^3^ per year for acetabular liners [40]. It has been suggested that with these low wear rates, any aseptic revision may be more related to the fixation of the components rather than to bearing wear [18]. One of the disadvantages of the ceramic combination is the potential for ceramic fracture, which has been reported to range from 0.01 to 3.5% [41–43]. In a meta-analysis of 10,571 THAs in 45 studies, Yoon et al. found the rate of ceramic fracture at postoperative 2.0 to 18.8 years was 0.5% in the forte group and 0.2% in the delta group (*p* = .059). Per 1000 patient years, the rate of ceramic fracture was 0.9 in the forte group and 0.5 in the delta group (*p* = .072). The authors found no significant associations between incidence of fracture and length of postoperative time, patient age, or BMI [44].

## Conclusion

The improvements in baseline scores and low complication rate provide support for use of the R3 BIOLOX delta Ceramic Acetabular System for patients undergoing primary THA.

## Data Availability

All data generated or analyzed during this study are included in this published article. The datasets used and/or analyzed during the current study are available from the corresponding author on reasonable request.

## Abbreviations

AOANJRR: Australian Orthopaedic Association National Joint Replacement Registry
BMI: Body mass index
CoC: Ceramic-on-ceramic
CoXPE: Ceramic-on-highly cross-linked polyethylene
mHHS: Modified Harris hip score
MMRM: Mixed model for repeated measures
MoM: Metal-on-metal
MoP: Metal-on-polyethylene
NJR: National Joint Registry of England, Wales, Northern Ireland and the Isle of Man
SD: Standard deviation
THA: Total hip arthroplasty
UCLA ARS: University of California, Los Angeles Activity Rating Scale
WOMAC: Western Ontario and McMaster Universities Osteoarthritis Index
